# Crater–Spectrum Feature Fusion Method for *Panax notoginseng* Cadmium Detection Using Laser-Induced Breakdown Spectroscopy

**DOI:** 10.3390/foods13071083

**Published:** 2024-04-01

**Authors:** Rongqin Chen, Xiaolong Li, Weijiao Li, Rui Yang, Yi Lu, Zhengkai You, Fei Liu

**Affiliations:** 1 College of Biosystems Engineering and Food Science, Zhejiang University, Hangzhou 310058, China; chenrq@zju.edu.cn (R.C.); xiaolli@zju.edu.cn (X.L.); ryang@zju.edu.cn (R.Y.); yilu@zju.edu.cn (Y.L.); youzk@zju.edu.cn (Z.Y.); 2 School of Chinese Material Medica, Yunnan University of Chinese Medicine, Kunming 650500, China

**Keywords:** signal uncertainty, crater morphology compensation, characteristic peak ratio correction, rapid detection, toxic metal pollution

## Abstract

*Panax notoginseng* (*P. notoginseng*) is a valuable herbal medicine, as well as a dietary food supplement known for its satisfactory clinical efficacy in alleviating blood stasis, reducing swelling, and relieving pain. However, the ability of *P. notoginseng* to absorb and accumulate cadmium (Cd) poses a significant environmental pollution risk and potential health hazards to humans. In this study, we employed laser-induced breakdown spectroscopy (LIBS) for the rapid detection of Cd. It is important to note that signal uncertainty can impact the quantification performance of LIBS. Hence, we proposed the crater–spectrum feature fusion method, which comprises ablation crater morphology compensation and characteristic peak ratio correction (CPRC), to explore the feasibility of signal uncertainty reduction. The crater morphology compensation method, namely, adding variables using multiple linear regression (MLR) analysis, decreased the root-mean-square error of the prediction set (RMSEP) from 7.0233 μg/g to 5.4043 μg/g. The prediction results were achieved after CPRC pretreatment using the calibration curve model with an RMSEP of 3.4980 μg/g, a limit of detection of 1.92 μg/g, and a limit of quantification of 6.41 μg/g. The crater–spectrum feature fusion method reached the lowest RMSEP of 2.8556 μg/g, based on a least-squares support vector machine (LSSVM) model. The preliminary results suggest the effectiveness of the crater–spectrum feature fusion method for detecting Cd. Furthermore, this method has the potential to be extended to detect other toxic metals in addition to Cd, which significantly contributes to ensuring the quality and safety of agricultural production.

## 1. Introduction

*Panax notoginseng* (*P. notoginseng*) is a valuable medicinal plant in great demand. Its root, acknowledged as a medicinal part, is beneficial for blood circulation, blood stasis alleviation, detumescence, and pain alleviation in clinical practice [1]. Modern pharmaceutical research hypothesizes that *P. notoginseng* can also be used for the treatment of cardiovascular diseases, hypertension, and hyperlipidemia. *P. notoginseng* is available as a dietary food supplement and healthcare product due to its bioactive compounds, such as saponins and flavonoids [2]. The efficacy of *P. notoginseng* is affected by toxic metal contamination, a topic of increasing interest because of the abundance of mineral resources in *P. notoginseng*-cultivated soil. Cadmium (Cd) absorption and enrichment in *P. notoginseng* is relatively strong, and Cd is more easily transferred to the ground [3]. Cd contamination not only reduces the yield of *P. notoginseng* and diminishes the accumulation of bioactive compounds [4], but it also poses risks to environmental pollution and human health [5]. Consequently, excessive Cd pollution has emerged as a major concern, highlighting the critical importance of Cd detection for ensuring the quality and safety of *P. notoginseng* [6,7]. The regulated detection methods include atomic absorption spectrometry (AAS) [8,9] and inductively coupled plasma–mass spectrometry (ICP-MS) [10,11,12]. These methods are widely recognized due to their good repeatability, low detection limit, and high accuracy. However, samples need to undergo processes such as digestion and dilution to meet the requirements of these instruments. Their shortcomings are also apparent, with complex pretreatments, high time costs, and the requirement of professional operators, meaning they cannot ensure intelligent and rapid detection with a short response time.

Laser-induced breakdown spectroscopy (LIBS), an atomic emission spectroscopy depending on plasma formation, has the advantages of no or minimal pretreatment, a rapid detection process, and a wide analytical range, and it can be used in multiple elements, long-distance transmission, and online detection. The emission lines of LIBS that characterize the substance’s features can be applied for qualitative and quantitative analysis in coal production [13], agriculture [14,15], soil [16], and so on. Cd detection using LIBS has been researched for use in lots of plants, such as cabbage [17], herbs [18,19], lettuce [20], and rice [21]. The bottleneck that causes a relatively lower measurement precision and accuracy of LIBS toxic metal quantification is signal uncertainty, which hinders further development [22]. The factors influencing uncertainty come from various aspects, including the matrix effect, the LIBS system, and the surrounding environment.

Research on reducing signal uncertainty comprises sample preparation, system setting, and data processing. Yang et al. [23] proposed a solid–liquid–solid transformation method, with rice samples prepared by means of ultrasound-assisted extraction for Cd and Pb determination using LIBS. Wang et al. [24] optimized the laser energy and delay time to obtain spectra and then built a multiple linear regression model for Pb and Cu detection in *Ligusticum wallichii* with limits of detection of 15.7 and 6.3 μg/g, respectively. From the point of view of data processing, normalization [25,26] (based on the specific element [27], background [28], and peak area [29], etc.), calibration-free LIBS (CF-LIBS) [30,31], and multivariate analysis [32] have been used and have obtained improved results. Zhao et al. [33] detected five metal elements in lily bulbs using partial least squares regression (PLSR) by combining various data preprocessing and selection methods to build the best-fitting model. In comparison, Su et al. [34] adopted a framework that removed noise and low-intensity variables and then combined it with PLSR to simultaneously and quantitatively measure several toxic metals in *Sargassum fusiforme*. Nonetheless, the preparation-modified method requires more operations, and the data-driven model, based on multiple spectra information, fails to achieve a reduction in signal uncertainty, because the multivariate model is only partially able to compensate for signal uncertainty. Matrix effects refer to differences in the physical (particle size and distribution) and chemical (composition of elements) properties of the samples, which affect the generation and evolution of plasma [35]. Borduchi [36] and Lei [37] tried to reduce the matrix effects for soybean leaf and milk powder, respectively. CF-LIBS was employed in their studies, and this approach did not require the building of models when needed to meet specific conditions. The ablated crater can also reflect the laser ablation status and help researchers to understand the ablation process, so it is regarded as a plasma parameter, besides the line intensity and temperature [38]. Regarding the research on the relationship between signal intensity and crater morphology [39], energy optimization based on craters [40] and LIBS signal enhancement interpretation [41] has been carried out. Sun et al. [42] corrected the LIBS signal with the ablation crater volume to improve the signal’s repeatability. The above attempts demonstrate the vital roles craters play in LIBS analysis. However, existing studies on crater analysis have typically been carried out under different system parameters or utilized standard metal samples, which enlarge crater discrepancies and minimize sample variance.

**Figure 1:**
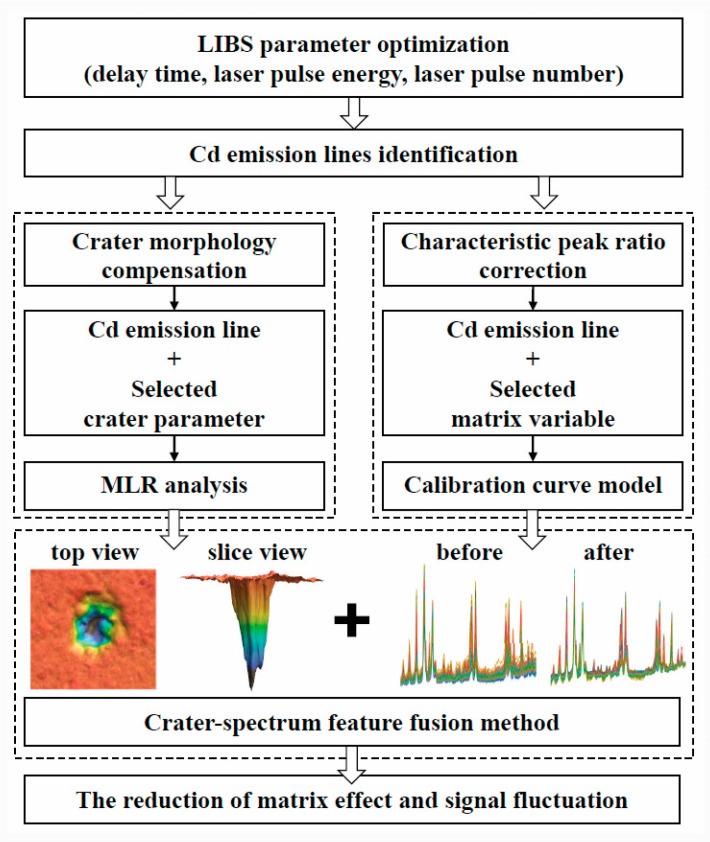
Flowchart depicting two approaches for matrix effect reduction.

In our study, *P. notoginseng,* sourced from various origins with diverse compositions, was used as the experimental material. The differences observed in the ablation craters prompted us to investigate the potential of combining LIBS raw signals and crater morphology to predict Cd concentrations. Following this, a simple framework known as the characteristic peak ratio correction (CPRC) method was proposed to refine the LIBS signals. Additionally, the crater–spectrum feature fusion method was employed to further enhance the analysis. As depicted in Figure 1, we aimed to develop a more robust model by addressing signal uncertainties from two perspectives: ablation crater morphology compensation and signal ratio correction.

## 2. Materials and Methods

### 2.1. Sample Preparation

Six brands of *P. notoginseng* powders were purchased from different sources at markets. Detailed information is shown in Table 1. Implementation standards reflect the difference in processing methods before the products entered the markets for sale. The quality of *P. notoginseng* powders varies due to differences in habitat, maturity, and processing methods, even though they are all derived from *P. notoginseng*. This variation results in a matrix effect during analysis. We first prepared a 0.01 mol/L cadmium nitrate solution by dissolving cadmium nitrate tetrahydrate (Sinopharm Chemical Reagent Co., Ltd., Shanghai, China), and then added different volumes to mix with 4 g of dried *P. notoginseng* powder, creating Cd-contaminated samples. To ensure homogeneity in the mixture, we added deionized water to create a suspension of *P. notoginseng*. After thorough stirring with a glass rod, the mixture was placed in an oven at 80 °C for 48 h to remove moisture.

The contaminated *P. notoginseng* powders were prepared at concentrations of 0, 0.5, 1, 10, 20, 30, 40, 50, 60, and 70 μg/g, resulting in ten processing levels. Then, 0.2 g contaminated powders were pressed into tablets with a diameter of 13 mm and thickness of 1 mm using a tablet machine at a pressure of 20 MPa for twenty seconds. Four samples were prepared for each concentration. Finally, 40 samples for each brand were collected. We randomly divided the 40 samples of each brand into four groups, labeled Group1, Group2, Group3, and Group4. Group1, with six brands, made up Dataset1. Dataset2, Dataset3, and Dataset4 were composed in the same way. Dataset1, Dataset2, and Dataset3 were used as the calibration set, and Dataset4 was used as the prediction set.

### 2.2. The Determination of Cd Reference Value

Considering the Cd concentration of the sample itself and the manipulation error, the Cd reference value was measured by means of ICP-MS (Agilent 7800ICPMS, Santa Clara, CA, USA) [43]. Samples needed to be pretreated before testing. Here, 0.1 g of contaminated powder from each sample was weighed and put into a digestion tubes; 5 mL of 65% nitric acid was added into each digestion tube. Then, the digestion tubes were placed in the graphite digestion furnace at 110 °C. After the sample had been nearly digested, the lids of the digestion tubes were opened to add the acid. When the above operations were completed, the digestion solution was placed in a volumetric flask and diluted with deionized water to 25 mL. Then, 5 mL of filter solution was used for the determination of the Cd concentration. Similar steps were described by Geng et al. [44]. The Cd reference value is shown in Table 2.

### 2.3. Experimental Instruments

#### 2.3.1. LIBS Instrumentation

**Figure 2:**
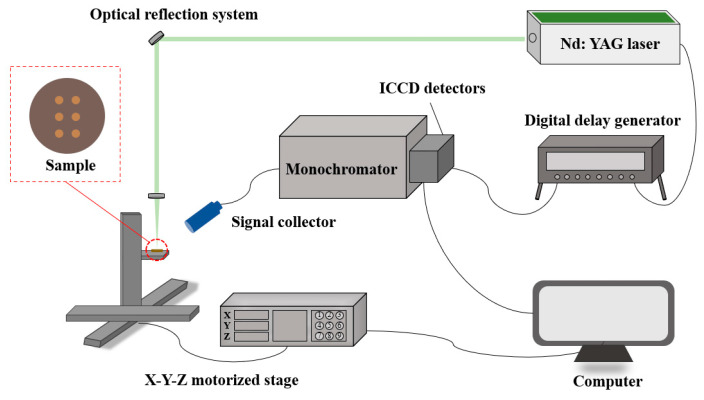
The schematic diagram of the LIBS system.

A self-assembled LIBS system was employed for LIBS data acquisition. As shown in Figure 2, a Q-switched Nd: YAG pulsed laser (Vlite-200, Beamtech Optronics, Beijing, China) generated the 532 nm laser with a pulse duration of 8 ns. The laser was focused on the samples using an optical reflection system. The electromagnetic signal of plasma radiation that was generated after the samples had been ablated was captured through a signal collector. The monochromator (SR-500i-A-R, Andor, Belfast, UK) was used to disperse light, and then an ICCD detector (iStar DH334T-18F-03, Andor, Belfast, UK) converted the optical signal into an electrical signal, presented in the computer. In this experiment, the LIBS spectra were collected in the range of 210-231 nm. A digital delay generator (DG645, Stanford Research Systems, Sunnyvale, CA, USA) was adopted to control the timings of lasers and ICCD detectors, and the laser frequency was set to 1 Hz. The LIBS system was detected via single-shot scanning. The sample was positioned on the X-Y-Z motorized stage (Zolix, Beijing, China), which was utilized to collect spectra at different sites. Before the experiment, several parameters were optimized, including a delay time of 2 μs, a gate width of 8 μs, and a laser pulse energy of 40 mJ. The laser ablation path was configured as a 2 × 3 array of craters. Each position underwent nine ablations, and the resulting spectra were averaged to minimize laser-induced point-to-point fluctuations. The laser beam was focused 2 mm below the sample surface to ensure stable signal acquisition [45].

#### 2.3.2. Ablation Crater Measurement

The shape measurement laser microscopy system, mainly consisting of a controller (VK-X1000, Keyence, Osaka, Japan) and measurement module (VK-X1050, Keyence, Osaka, Japan) and a base (VK-D1, Keyence, Osaka, Japan), was used to obtain the morphologies and parameters of craters. The VK-X1050 was equipped with a red semiconductor laser at a wavelength of 661 nm. The optical receiving elements included a 16-bit induction photomultiplier and an ultra-high fine color complementary metal oxide semiconductor (CMOS). The instrument found the samples’ focal lengths for each point by way of progressive scanning and pinhole conjugate focusing. The morphologies of different heights were obtained by physically moving the objective lens. The 3D morphology of the sample was recorded by means of longitudinal splicing.

### 2.4. Characteristic Peak Ratio Correction

We proposed a signal correction method that aimed at reducing the fluctuation of target emission lines, named the characteristic peak ratio correction method (CPRC). The specific steps of CPRC are as follows, and Cd was the target element.

(i) We obtained *n* LIBS spectra matrices *X* = [*x*_1_, …, *x_p_*]*_n_*_∗*p*_ (*p* is the number of wavelengths, regarded as *p* variables) and *n* Cd reference value matrices *Y* = *C_n_*. The characteristic peaks were Cd emission lines employed for analysis, referring to the National Institute of Standards and Technology (NIST) atomic spectral database. The *j*th (*j* = 1, 2, 3, …, *p*) wavelength position was selected successively from *X*, and the corresponding signal intensity was marked as *z_j_* (*z_j_* ∈ *X*). We then calculated the ratio of the characteristic peak intensity to *z_j_*:

*B_j_* = *x_N_*/*z_j_* (1)

where *x_N_* is the intensity of the characteristic peak, and *z_j_* is each variable of the spectrum.

(ii) We then calculated the linear correlation coefficient (*r*-value) between n *B_j_* and *C_n_*:

(2) $$r_{j}=\frac{\sum_{i=1}^{n}\left(B_{ji}-\bar{B_{j}}\right)\left(y_{i}-\bar{y}\right)}{\sqrt{\sum_{i=1}^{n}{\left(B_{ji}-\bar{B_{j}}\right)}^{2}}\sqrt{\sum_{i=1}^{n}{\left(y_{i}-\bar{y}\right)}^{2}}}$$

where $B_{ji}$ is the corrected intensity based on the *j*th wavelength position of the *i*th (*i* = 1, 2, 3, …, *n*) collection site; *y_i_* is the Cd reference value of *i*th collection site; $\bar{B_{j}}$ and $\bar{y}$ are the average values of signal intensity and Cd reference value, respectively.

The R-value was used as an evaluation index to find the highly correlated corrected intensity ($B_{ji}$) [46]. The higher the *r*-value, the more effective $B_{ji}$ is. Each variable (*z_j_*) that composes the LIBS spectrum participates in the trial (Equation (1)). The wavelength range of LIBS spectrum is composed of *p* wavelength numbers; thus, *p* calculated *r* values are obtained and arranged in descending order. The variables corresponding to the top *r* values are chosen as those that have a positive effect on the signal correction. The first *m* (*m* ≤ 10) variables of combination [*z_i_*]*_n_* are selected, and we here define these variables as matrix-related variables, as we thought these variables contributed to reducing the matrix effect, recorded as *Z* = [*z*_1_, …, *z_m_*]*_n_*. The mean of matrix-related variables is calculated as follows:

(3) $$\bar{Z}=\frac{1}{m}\sum_{1}^{m}z$$

where *m* is the number of matrix-related variables selected.

(iii) Output the corrected spectral matrix. The corrected spectral matrix is the ratio of *X* to $\bar{Z}$:

(4) $$X^{\prime }={\left[\frac{x_{1}}{\bar{Z}},\ldots ,\frac{x_{p}}{\bar{Z}}\right]}_{n*p}$$

where *x*_1_, …, *x_p_* are raw intensity values in the LIBS spectrum.

The approach to confirming *m* was as follows. We introduced variables one by one in order of *r* value from high to low. PLSR was performed based on the full spectrum that had been corrected. The RMSE of cross-validation is set as the evaluation index [47]. A combination of variables corresponding to the minimum RMSE was selected.

Corrected characteristic peaks can be selected and extracted from the new spectral matrix according to the wavelengths that they lie in, which are used for Cd concentration analysis.

### 2.5. Performance Evaluation and Software

We had to use evaluation indexes to check the model’s feasibility. In this study, the calibration results were evaluated with the determination coefficient (*R*^2^) and the root-mean-square error (RMSE). As the following formulas show, the closer *R*^2^ is to 1, the better the model performs. A lower RMSE indicates a smaller deviation between the predicted and reference values.

(5) $$R^{2}=1-\frac{\sum_{i=1}^{n}(\hat{y_{i}}-y_{i}){\;}^{2}}{\sum_{i=1}^{n}(\bar{y_{i}}-y_{i}){\;}^{2}}$$

(6) $$RMSE=\sqrt{\frac{1}{n}\cdot \sum_{i=1}^{n}{\left(y_{i}-\hat{y_{i}}\right)}^{2}}$$

Here, $\hat{y_{i}}$ and $y_{i}$ are the predicted values and the reference value of the *i*-th observation, respectively. $\bar{y_{i}}$ is the average of the true value of the *i*-th observation. *n* is the total number of observations.

The ablation crater’s profile was measured using MultiFileAnalyzer 2.2.0.93 (Keyence, Osaka, Japan). Data analysis in this study was performed in MATLAB 2019b (The MathWorks, Natick, MA, USA).

## 3. Results and Discussions

### 3.1. Spectral Profile

**Figure 3:**
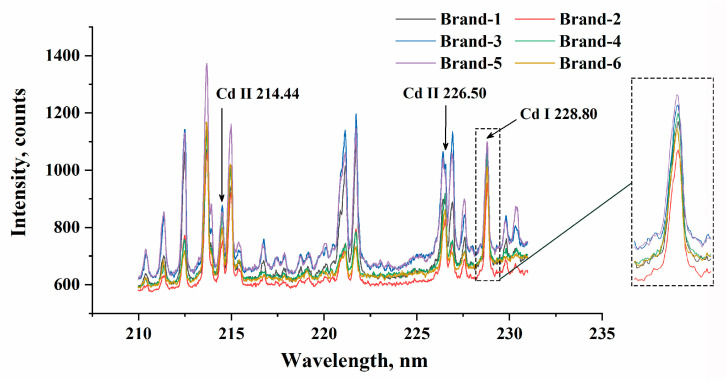
The average spectra of *P. notoginseng* with the highest Cd concentrations.

Figure 3 displays the average spectra of the various ablation sites on *P. notoginseng* tablets, with the highest Cd concentrations from each brand in Dataset1 chosen as representatives. By referencing the NIST database, we identified several high-intensity spectral lines. Three Cd emission lines were pinpointed at wavelengths of 214.44, 226.50, and 228.80 nm. These three Cd emission lines are commonly utilized as analytical lines for Cd detection [17,19,21]. In addition, some other emission lines with higher intensity were also identified and are listed in Table 3. The relatively pure peaks of Cd II 214.44 nm and Cd I 228.80 nm could be recognized. The peaks of Cd II 226.50 nm were influenced by other element emission lines, such as Fe II 226.48 nm and Fe I 226.50 nm. The spectral peaks of the various brands of *P. notoginseng* powders were basically the same. The differences in signal intensity and trends at some wavelengths arose due to signal uncertainty. In the enlarged image of Cd I 228.80 nm, signal fluctuation and background drifting can apparently be observed.

According to the principle of LIBS, a narrow line dominates corresponding to the element, and the line intensity is proportional to the atomic concentration [48]. The intensity of the element emission line goes up with the concentration when the interference is small, showing a linear relationship [49]. To verify the difference in the models’ performances between a single brand and various brands, we analyzed the emission lines of Cd based on the calibration curve model.

**Figure 4:**
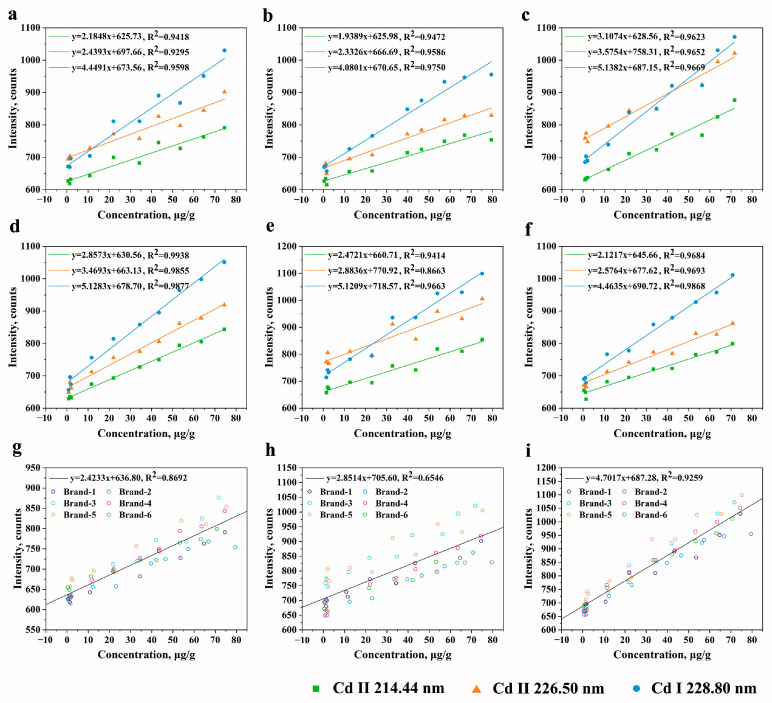
Comparison of *P. notoginseng* spectral intensities with different Cd concentrations from various brands: (**a**) Brand-1; (**b**) Brand-2; (**c**) Brand-3; (**d**) Brand-4; (**e**) Brand-5; (**f**) Brand-6. Comparison of *P. notoginseng* spectral intensities with different Cd concentrations from six brands: (**g**) of Cd II 214.44; (**h**) Cd II 226.50 nm; (**i**) of Cd I 228.80 nm.

As can be seen in Figure 4, the intensity was plotted at the wavelengths of Cd II 214.44 nm, Cd II 226.50 nm, and Cd I 228.80 nm for various brands. Figure 4a–f present the results of the fitting of experimental data for Brand-1, Brand-2, Brand-3, Brand-4, Brand-5, and Brand-6, respectively. It can be seen that the relationship between the LIBS intensity and Cd concentration is almost linear, and the fitting performances of *P. notoginseng* at the three emission lines are different. There is strong linearity among single brands of *P. notoginseng*. Even the results for Brand-4 and Brand-6 are satisfactory, with *R*^2^ of 0.98. Figure 4g–i show the linear fitting of data from the six brands at the wavelengths of Cd II 214.44 nm, Cd II 226.50 nm, and Cd I 228.80 nm, respectively. However, the fitting results are poor when the experimental data from the six brands are put together. Therefore, the signal uncertainty arising from matrix effects inevitably diminishes the linear correlation between signal intensity and Cd concentration. It is imperative to address signal uncertainty reduction in order to achieve more accurate results. This improvement is essential when constructing a model capable of predicting Cd concentrations simultaneously across six different brands.

### 3.2. Principal Component Analysis

**Figure 5:**
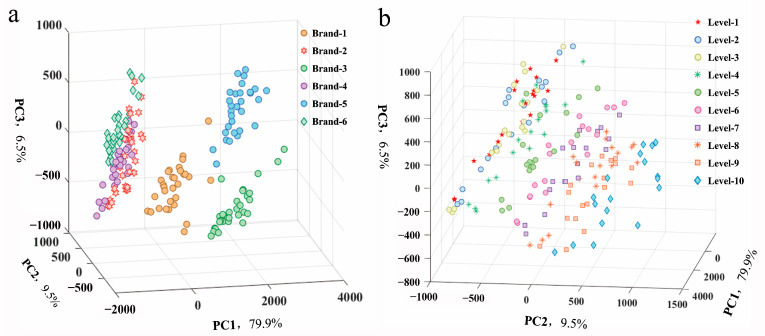
3D scatter plot of *P. notoginseng* powder based on the first three principal components (PCs): (**a**) six brands; (**b**) ten processing levels.

Principal component analysis (PCA) was applied to display the distribution and separability among all samples. Two 3D scatter plots from six brands and ten processing levels of *P. notoginseng* powder are displayed in Figure 5. The PCA results show that the first three principal components (PC) (79.9% for PC1, 9.5% for PC2, and 6.5% for PC3) explained 95.9% of the total variance in the LIBS spectra. Figure 5a shows the classification among various brands. Four distinction zones can be seen, and Brand-1, Brand-3, and Brand-5 cluster separately. Brand-2, Brand-4, and Brand-6 have different clustering centers, but they overlap, making them difficult to distinguish. From the qualitative point of view, Figure 5a illustrates that there were discrepancies among the various brands. Figure 5b presents the classification of ten processing levels. Distinct clustering zones failed to form. Overall, samples from the same source tended to cluster together, while samples with similar concentrations struggled to cluster. This indicates that samples are more influenced by differences in the matrix composition due to different sources. Therefore, it is necessary to reduce matrix effects and improve detection accuracy. The detection of concentrations among various brands requires further analysis.

### 3.3. The Crater Morphology Analysis

**Figure 6:**
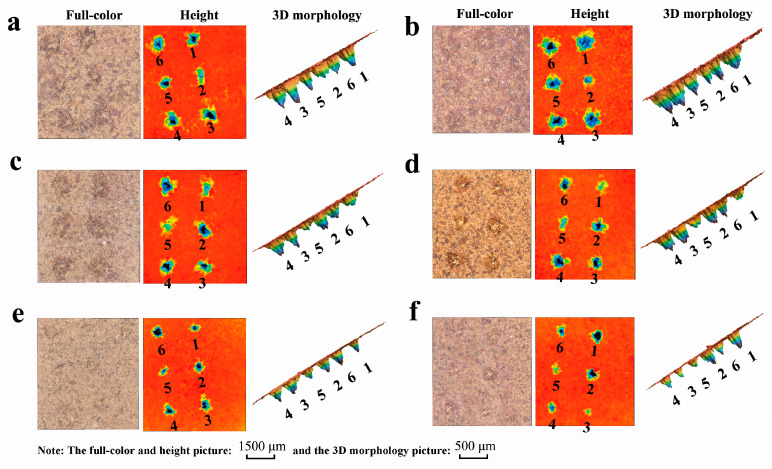
The full-color pictures, height pictures, and 3D morphology pictures of six ablated positions’ craters: (**a**) Brand-1; (**b**) Brand-2; (**c**) Brand-3; (**d**) Brand-4; (**e**) Brand-5; (**f**) Brand-6.

*P. notoginseng* powders from six brands with different physical properties yielded to different crater morphologies. Figure 6 displays the full-color pictures, height pictures, and a 3D morphology picture of six ablated positions’ craters, with the highest Cd concentration in Dataset1 being representative. In order to present the pictures more comprehensively and clearly, we set different scaling ratios and placed the reference scales on the figure. In the full-color pictures, we can see that the color of each brand is not the same. The *P. notoginseng*’s color in Figure 6a,c,e is close to brown; in Figure 6b,f, it is close to red; and in Figure 6d, it is close to yellow. The crater sizes of Brand-1 and Brand-2 are bigger than the others due to their looser texture. Brand-5, with the densest texture, presents the smallest damage regions. More loosely textured *P. notoginseng* powders resulted in more damage and increased laser penetration, which brought about larger craters.

To further illustrate the differences in craters as influenced by the matrix, the ablation crater profile was measured. We set the upper surface of the tablet as a threshold. The analyzer system could calculate nine parameters of craters, including volume, cross-sectional area, surface area, average depth, maximum depth, perimeter, horizontal Feret’s diameter, vertical Feret’s diameter, and circle equivalent diameter, which together characterize the craters’ morphologies. The volume and surface area refer to the three-dimensional space enclosed by the shape and threshold of the measurement object. The cross-sectional area was obtained by crosscutting the cross-sectional area of the shape with a threshold. The average height and maximum height represent the average and maximum deviation between the height and the threshold of the shape, respectively. The perimeter, horizontal Feret’s diameter, vertical Feret’s diameter, and circle equivalent diameter describe the sample surface states that are ablated by the application of a laser from various perspectives. The distances between the parallel lines of the upper and lower boundaries of the ablated surface are defined as the vertical Feret’s diameter, and the left and right boundaries are defined as the horizontal Feret’s diameter. The circle equivalent diameter is the diameter of a circle whose area is equal to its cross-sectional area. Table 4 shows nine measurement values among six brands of *P. notoginseng* powders in Dataset1. We can identify significant discrepancies, with RSD beyond 20%. As a result, the ablation crater profiles of each brand with different physical properties vary. The sample matrix affects the ablation process, which reflects the interaction between the sample and the laser, itself causing LIBS signal fluctuations and crater profile differences. This finding inspired us to include crater profiles as variables when analyzing Cd concentration to reduce the signal uncertainty that rises during concentration prediction.

### 3.4. Quantitative Analysis of Cadmium

#### 3.4.1. Crater Morphology Compensation Method

The linear quantitative analysis method was applied in combination with LIBS to achieve Cd detection in relation to the linear fitting in Figure 4. Hence, the calibration curves were constructed, and multiple linear regression (MLR) analysis was performed. The calibration curve was drawn with the Cd concentration as the *X*-axis and the LIBS intensity as the Y-axis, with a single *X*-variable. A linear function was applied to fit the experimental data, expressed as *y* = *ax + b*. MLR involves regressing one *Y*-variable on a set of *X*-variables [45,50]. The nine parameters of craters and three Cd lines of LIBS signals were the independent variables used as inputs, and the Cd concentrations were the dependent variable used as the output. MLR was also conducted to explore the degrees of contribution of crater morphologies and LIBS signals. The paired *t*-test was used to evaluate whether the independent variable has a significant linear influence on the dependent variable. If the *p*-value is <0.05, the observed effect is not due to random variations. Thus, the variables under study have a significant effect. Stepwise regression analysis was carried out to select contributing variables and optimize the regression model [51].

**Figure 7:**
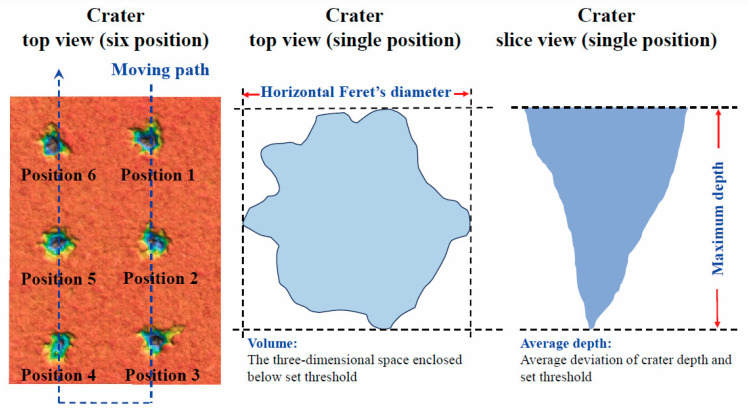
The schematic illustration of selected crater parameters.

We input twelve parameters, including crater parameters and emission lines, into the initial model so as to make good use of crater profiles. Then, one *X*-variable with the highest *p*-value, larger than 0.05, was set to be removed every time we utilized MLR based on the new *X*-variable combinations until the *p*-values for all *X*-variables were below 0.05. The variable selection process is presented in Table 5. We eliminated the variables without significant effects on *Y* one by one to obtain the best model with the lowest RMSE. Finally, the volume, average depth, maximum depth, and horizontal Feret’s diameter of craters were recognized as *X*-variables in the following analysis, thus together contributing significantly to the linear model. The selected craters’ parameters are marked in Figure 7 to provide straightforward information, which is roughly consistent with the findings of other studies [52]. The blue line indicates the moving path of the *X*-*Y*-*Z* motorized stage. The pulsed laser beam ablates mass from a sample surface. Thus, the volume indirectly reflects the ablated mass quantity [53]. The average depth and maximum depth describe the penetration of the laser through the sample [54]. The horizontal Feret’s diameter characterizes the crater shape of the sample surface [55]. These selected parameters were sufficiently applied to compensate for the signal uncertainty.

In this part, the effect of crater compensation on the single-point signal was explored, which did not involve other variables. Therefore, the raw signal was not preprocessed. Linear regression analysis results based on different variables, peak intensity, or selected crater parameters are shown in Table 6. A single emission line, set as the variable, was analyzed using a calibration curve model. The feasibility of improving detection capacity by combining the LIBS signal with selected crater parameters was explored based on MLR. Cd I 228.80 nm exhibits a stronger correlation with Cd concentration compared to other Cd emission lines. This could be attributed to the absence of significant interference peaks near 228 nm in the *P. notoginseng* powders. A better performance was obtained with higher R^2^ and lower RMSE when adding variables of craters for analysis, compared with using only LIBS peak information. Therefore, the result implies that the crater morphology caused by laser ablation compensates for the fluctuation of a single peak and thus enhances prediction ability, regardless of whether it is influenced by the interference of other elements or a poor correlation with Cd concentration. The combination of three emission lines and the selected crater parameters achieved the lowest RMSE, with an RMSEC of 5.6434 μg/g and an RMSEP of 5.4043 μg/g. Additionally, we utilized multiple emission lines as inputs to investigate whether increasing the number of emission lines could enhance the predictive effectiveness. The findings indicate that augmenting the number of emission lines enhanced the model’s performance. Hence, crater profiles play a role in predicting Cd concentrations, and compensating for signal uncertainty through ablation crater parameters is a viable approach.

#### 3.4.2. Characteristic Peak Ratio Correction (CPRC) Method

A stable LIBS signal with less uncertainty interference after correction is conducive to enhancing accuracy and sensitivity. Thus, CPRC has been further carried out to achieve more ideal results. The wavelengths selected as matrix variables from the calibration set are shown in Table 7, the variable numbers of which are less than ten. The position of the selected wavelength is almost at the end of the spectrum, which reduces the interference of background signal and background drifting from the matrix and experimental conditions. Considering the spectral profiles shown in Figure 3, two commonly used preprocessing methods, baseline correction and normalization, were applied for comparison. Baseline corrections with asymmetric least squares smoothing [56] and total area normalization [57] were performed.

The results based on the calibration curve model can be seen in Table 8; we can see that total area normalization pretreatment obtained better results than no pretreatment at the wavelength of Cd II 226.50 nm and at Cd I 228.80 nm to some extent. The baseline correction showed worse results than no pretreatment. The proposed CPRC method improved the analytical performance the most. The standard deviation of the data set was significantly cut down, resulting from the reduced signal fluctuation. The RMSEP values at the three emission lines reached 4.5823, 5.7979, and 3.4980 μg/g, respectively. We could infer that the spectral deviation in our study is influenced more by the matrix effect than by the baseline drift of the instrument.

**Figure 8:**
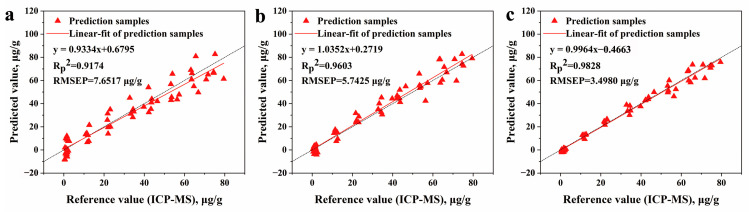
The relationship between the reference value and the predicted value measured using a calibration curve based on Cd I 228.80 nm (**a**) without pretreatment, (**b**) with normalization and (**c**) with CPRC.

The samples after CPRC pretreatment in both the calibration set and prediction set obtained the best performance. The most optimal results using Cd I 228.80 nm as a variable with different pretreatments based on the calibration curve model are presented in Figure 8. By referencing the calculation method in [58], we can see that the limit of detection was 1.92 μg/g and the limit of quantification was 6.41 μg/g. The relationship between the reference value and predicted value shows that the distribution is closest to the linear fit lines with CPRC pretreatment, which demonstrates that CPRC is beneficial to signal intensity correction when seeking to obtain better sensitivity. The acceptable performance of CPRC may be due to the processing, as a result of which it takes into account the important contribution of emission lines to the model, while normalization pretreatment ignores the contribution of characteristic variables and merely makes an undifferentiated correction based on the full spectrum. In short, CPRC reduced the number of variables considerably and achieved better results in the confirmatory study.

Partial least squares regression (PLSR), as a mode of multivariate analysis, is considered an effective tool to deal with the matrix effect by extracting useful information in such a way that it outperforms univariate analysis [59,60]. In this study, PLSR based on a full spectrum was performed with different pretreatments to evaluate the effectiveness of CPRC again. The peak intensity of emission line 228.80 nm showed the best ability to predict Cd concentration with the highest *R*^2^. Thus, the Cd I 228.80 nm is applied as a characteristic peak in the CPRC method to correct the full spectrum. From Table 8, we can see that the data without pretreatment based on the PLSR model could reach an acceptable outcome with an *R*^2^ above 0.98, which is achieved by considering more effective variables using full-spectrum information. The CPRC method further improved the results, with the lowest RMSEP of 3.1889 μg/g. The satisfactory results indicate that the proposed CPRC contributes to the linearity and accuracy of calibration models. Also of note is that the prediction results based on the calibration curve model with CPRC are almost as good as those with PLSR, but with reduced complexity of the model. The variables required for PLSR are also not small. CPRC requires only a few variables, usually less than ten, to achieve spectrum correction, which lowers the need for variables and enhances computational efficiency. CPRC shows advantage and application potential in developing portable instruments wherein the number of variables available is limited.

#### 3.4.3. Crater–Spectrum Feature Fusion Method

The feature information extracted from the crater morphology and the corrected LIBS signal were fused. A single LIBS signal (Cd I 228.80 nm as representative) and multiple LIBS signals were combined with crater parameters. The crater parameters selected in Section 3.3 and the corrected intensities were used as the input variables, and Cd concentrations were output for analysis using MLR. As is shown in Table 9, the results were improved when the corrected signal intensity using CPRC replaced the raw intensity to carry out MLR analysis (compared with Table 6).

Meanwhile, we input the combination of variables into the non-linear model, with the intention of studying the performance of non-linear models established on fused data sets. Two typical non-linear models, least square support vector machine (LSSVM) [61] and random forest (RF) [62], were used to predict Cd concentration. Table 9 lists the modeling results with various variables and different models based on the crater–spectrum feature fusion method. The LSSVM model achieved the best results, with an R_P_^2^ of 0.9885 and an RMSEP of 2.8556 μg/g. Therefore, the crater–spectrum feature fusion method has demonstrated its effectiveness in model development. By combining crater morphology and LIBS at the feature level, more comprehensive information about the physical and chemical properties of samples can be obtained, enhancing the detection capability.

## 4. Conclusions

To tackle the challenge of signal uncertainty, especially in plant samples with complex compositions, we undertook a study to investigate the effectiveness of crater morphology compensation and signal intensity correction. Firstly, the crater parameters that characterize the states were carefully selected. By incorporating these crater parameters as input variables in MLR analysis, the RMSEP was reduced from 7.0233 μg/g to 5.4043 μg/g. This result proves that the crater morphology compensation method was conducive to constructing a model suitable for various categories of *P. notoginseng*. Secondly, the characteristic peak correction employed in data pretreatment was demonstrated to be effective for detecting Cd concentrations in *P. notoginseng*. The CPRC pretreatment exhibited an improved linear relationship between the reference value and the predicted value. Prediction results were obtained using the calibration curve model with an RMSEP of 3.4980 μg/g and using the PLSR model with RMSEP of 3.1889 μg/g. Thirdly, by integrating crater morphology compensation and the CPRC method, a crater–spectrum feature fusion method was proposed, which yielded satisfactory results in our study. This fusion method was applied to both linear and non-linear models, showing good practicality. The best result was derived when combining crater–spectrum feature fusion and the LSSVM model, with the lowest RMSEP of 2.8556 μg/g.

The proposed approaches are anticipated to advance the application of LIBS for toxic metal detection in plant samples. Crater morphology compensation, which accounts for variations in sample states, can help reduce deviations to a certain extent. The CPRC method is expected to gain widespread acceptance as a technique for preprocessing LIBS spectra in order to minimize signal variations, proving effective in both univariate and multivariate analyses. The crater–spectrum feature fusion method, being relatively straightforward and more precise, is suitable for use with portable LIBS instruments. Certainly, it is important to note that further improvements beyond our current study are necessary. Exploring the ability of the CPRC method to extend to multi-element synchronous correction is essential. The integration of crater morphology parameter calculation and the LIBS signal acquisition system could enhance the collection of more useful information for analysis. With ongoing developments in LIBS systems and data processing, the rapid and accurate detection of toxic metals in plant samples holds significant promise, ensuring the quality and safety of agricultural production.

## Figures and Tables

**Table 1:** Sample information from different brands.

Variety ID	Implementation Standard	Number of Samples
Brand-1	Chinese pharmacopoeia	40
Brand-2	Primary agricultural products	40
Brand-3	Primary agricultural products	40
Brand-4	Chinese pharmacopoeia	40
Brand-5	Primary agricultural products	40
Brand-6	Local pharmacopoeia	40

**Table 2:** Cd reference values of *P. notoginseng* powders, obtained by means of ICP-MS.

	Brand-1	Brand-2	Brand-3	Brand-4	Brand-5	Brand-6
Min. (μg/g)	0.57	0.45	0.98	0.83	1.52	0.48
Max. (μg/g)	74.52	79.52	71.71	74.47	75.20	70.80
Mean (μg/g)	30.70	32.88	30.70	30.72	31.33	29.90

**Table 3:** Main LIBS emission lines of *P. notoginseng* powder.

Elements	Wavelength (nm)
Al	221.00, 226.35, and 226.91
Si	212.41, 220.80, 221.09, 221.17, 221.67, and 221.81
Ca	211.28, 212.30, 215.08, and 227.55
Fe	213.65, 213.70, 215.24, 215.59, 221.10, 221.71, 226.48, 226.50, and 227.71

**Table 4:** Measurement values of ablation craters from the six brands in Dataset1.

Variable	Min.	Max.	Mean	RSD
Volume/µm^3^	6,948,531	312,237,208	59,940,633	76%
Cross sectional area/µm^2^	180,775	1,831,790	626,770	46%
Surface area/µm^2^	231,658	3,190,570	911,987	51%
Average depth/µm	28	179	88	32%
Maximum depth/µm	89	571	278	27%
Perimeter/µm	2083	13,829	5555	38%
Horizontal Feret’s diameter/µm	503	2034	1018	24%
Vertical Feret’s diameter/µm	517	2042	1049	23%
Circle equivalent diameter/µm	480	1527	873	22%

**Table 5:** The *p*-value of *X*-variables based on stepwise regression analysis.

Variable	1	2	3	4	5	6
Volume	0.2165	0.1311	0.0855	0.0974	0.0663	0.0086 *
Average depth	0.0285 *	0.0062 *	0.0014 *	0.0005 *	0.0000 *	0.0000 *
Maximum depth	0.0000 *	0.0000 *	0.0001 *	0.0001 *	0.0000 *	0.0000 *
Horizontal Feret’s diameter	0.0660	0.0479 *	0.0835	0.1098	0.1783	0.0373 *
Cd II 214.44 nm	0.0102 *	0.0102 *	0.0103 *	0.0086 *	0.0113 *	0.0123 *
Cd II 226.50 nm	0.0000 *	0.0000 *	0.0000 *	0.0000 *	0.0000 *	0.0000 *
Cd I 228.80 nm	0.0000 *	0.0000 *	0.0000 *	0.0000 *	0.0000 *	0.0000 *
Surface area	0.2031	0.1931	0.1786	0.2078	0.6127	
Cross-sectional area	0.4452	0.1368	0.2303	0.2296		
Perimeter	0.2745	0.2746	0.5054			
Vertical Feret’s diameter	0.3262	0.2883				
Circle equivalent diameter	0.9649					

*: The variable exerts a significant linear influence on the Cd concentration.

**Table 6:** Linear regression analysis with different variables.

Variable	Rc^2^	RMSEC (μg/g)	Rp^2^	RMSEP (μg/g)
Cd II 214.44 nm	0.8535	10.8314	0.8830	9.1792
Cd II 226.50 nm	0.6255	20.2269	0.6143	18.6068
Cd I 228.80 nm	0.9084	8.3028	0.9174	7.6517
Cd II 214.44 nm, Craters	0.8929	9.6902	0.9238	9.7984
Cd II 226.50 nm, Craters	0.7117	14.8176	0.7360	14.9050
Cd I 228.80 nm, Craters	0.9402	6.8812	0.9568	6.5869
Cd II 214.44 nm, Cd II 226.50 nm	0.8822	10.3789	0.9182	11.0419
Cd II 214.44 nm, Cd I 228.80 nm	0.9195	7.9063	0.9175	8.4123
Cd II 226.50 nm, Cd I 228.80 nm	0.9405	6.8192	0.9458	7.0339
Cd II 214.44 nm, Cd II 226.50 nm, Craters	0.9069	9.2159	0.9368	9.3580
Cd II 214.44 nm, Cd I 228.80 nm, Craters	0.9467	6.4911	0.9566	6.2532
Cd II 226.50 nm, Cd I 228.80 nm, Craters	0.9590	5.6944	0.9682	5.4291
Three Cd emission lines	0.9419	6.7512	0.9445	7.0233
Three Cd emission lines, Craters	0.9598	5.6434	0.9679	5.4043

**Table 7:** The selected variables used for characteristic peak correction.

Characteristic Peak	Num.	Selected Wavelengths as Matrix Variables (nm)
Cd II 214.44 nm	8	230.69; 230.58; 230.73; 230.66; 230.71; 230.81; 230.97; 230.83
Cd II 226.50 nm	5	229.85; 229.79; 229.87; 229.77; 229.75
Cd I 228.80 nm	8	230.73; 230.71; 230.75; 230.69; 230.97; 230.89; 230.87; 230.83

**Table 8:** Quantitative analysis results with different pretreatment methods.

Model	Pretreatment	Wav. (nm)	Rc^2^	RMSEC (μg/g)	R_P_^2^	RMSEP (μg/g)
Calibration curves	Raw	214.44	0.8535	10.8314	0.8830	9.1792
		226.50	0.6255	20.2269	0.6143	18.6068
		228.80	0.9084	8.3028	0.9174	7.6517
	Baseline	214.44	0.8473	11.0970	0.8084	11.6209
		226.50	0.6007	21.3171	0.5005	23.2237
		228.80	0.8608	10.5128	0.8890	12.6730
	Normalization	214.44	0.8856	15.3480	0.8771	14.4296
		226.50	0.8991	9.6649	0.9279	7.6563
		228.80	0.9612	6.1469	0.9603	5.7425
	CPRC	214.44	0.9714	4.4882	0.9706	4.5823
		226.50	0.9478	6.2345	0.9543	5.7979
		228.80	0.9820	3.5259	0.9828	3.4980
PLSR	Raw	Full	0.9834	3.4846	0.9831	3.9480
	Baseline	Full	0.9709	4.5606	0.9398	6.5590
	Normalization	Full	0.9851	3.3040	0.9874	3.3297
	CPRC	Full	0.9870	3.0715	0.9879	3.1889

**Table 9:** Modeling results based on crater–spectrum feature fusion strategy.

Model	Variables	R_C_^2^	RMSEC (μg/g)	R_P_^2^	RMSEP (μg/g)
MLR	Craters, Corrected Cd I 228.80 nm	0.9836	3.4913	0.9850	3.3126
	Craters, three corrected Cd emission lines	0.9848	3.3655	0.9865	3.1817
LSSVM	Craters, Corrected Cd I 228.80 nm	0.9851	3.2753	0.9872	2.9971
	Craters, three corrected Cd emission lines	0.9860	3.1630	0.9885	2.8556
RF	Craters, Corrected Cd I 228.80 nm	0.9903	2.8174	0.9834	3.6354
	Craters, three corrected Cd emission lines	0.9942	2.0747	0.9882	3.0091

## Data Availability

The original contributions presented in the study are included in the article, further inquiries can be directed to the corresponding author.
